# PD-L1 expression in liver metastasis: its clinical significance and discordance with primary tumor in colorectal cancer

**DOI:** 10.1186/s12967-020-02636-x

**Published:** 2020-12-11

**Authors:** Xiao-Li Wei, Xuan Luo, Hui Sheng, Yun Wang, Dong-Liang Chen, Jia-Ning Li, Feng-Hua Wang, Rui-Hua Xu

**Affiliations:** 1 Department of Medical Oncology, Sun Yat-Sen University Cancer Center, State Key Laboratory of Oncology in South China, Collaborative Innovation Center for Cancer Medicine, 651 Dong Feng Road East, Guangzhou, 510060 Guangdong China; 2 Department of Hepatobiliary Oncology, Sun Yat-Sen University Cancer Center, State Key Laboratory of Oncology in South China, Collaborative Innovation Center for Cancer Medicine, Guangzhou, 510060 China; 3 State Key Laboratory of Oncology in South China, Sun Yat-Sen University Cancer Center, Collaborative Innovation Center for Cancer Medicine, Guangzhou, 510060 China; 4 Department of Hematologic Oncology, Sun Yat-Sen University Cancer Center, State Key Laboratory of Oncology in South China, Collaborative Innovation Center for Cancer Medicine, Guangzhou, 510060 China; 5 Department of Clinical Trial Center, Sun Yat-Sen University Cancer Center, State Key Laboratory of Oncology in South China, Collaborative Innovation Center for Cancer Medicine, Guangzhou, 510060 China; 6 Research Unit of Precision Diagnosis and Treatment for Gastrointestinal Cancer, Chinese Academy of Medical Sciences, Guangzhou, 510060 China

**Keywords:** PD-L1, Liver metastases, Primary tumor, Colorectal cancer

## Abstract

**Background:**

The outcomes of immune checkpoint inhibitors in cancer patients with liver metastases are poor, which may be related to a different tumor microenvironment in liver metastases from primary tumors. This study was aimed to analyze PD-L1 expression and the immune microenvironment status in liver metastases and compare the differences of PD-L1 expression between primary tumors and liver metastases of colorectal cancer.

**Methods:**

74 cases of pathologically confirmed colorectal cancer with liver metastasis underwent resection from our hospital were included. Tissue microarrays were used for the interpretation of PD-L1 expression, cluster of differentiation 4 (CD4) and CD8 density by immunohistochemistry. We evaluated the disparity between primary tumor and liver metastasis in PD-L1 expression, CD4 and CD8 density and analyzed the factors associated with obvious PD-L1 disparity.

**Results:**

The expression of PD-L1 was positively related to the density of CD4 and CD8 in liver metastases. The expression of PD-L1 in liver metastases was higher than in primary tumors in certain subgroups, including patients with concurrent liver metastases (n = 63, *p* = 0.05), patients receiving concurrent resection of primary and metastatic tumors (n = 56, *p* = 0.04). The two subgroups generally reflected those without inconsistent external influences, such as treatment and temporal factors, between primary tumors and liver metastases. In these subgroups, the intrinsic differences of microenvironment between primary tumors and liver metastases could be identified. Furthermore, tumor differentiation [moderate vs. poor: *OR* = 0.23, 95% *CI*: 0.03–0.99, *p* = 0.05)] were demonstrated to be associated with obvious discordance of PD-L1 expression between primary tumors and liver metastases.

**Conclusions:**

The expression of PD-L1 in liver metastases was higher than in primary tumors in subgroups, reflecting intrinsic microenvironment differences between primary and metastatic tumors. Obvious discordance of PD-L1 expression between primary tumor and liver metastasis was significantly related to the tumor differentiation.

## Background

In recent years, the use of immune checkpoint inhibitors offered new hopes for cancer treatment [1]. With the use of PD-1/PD-L1 inhibitors in various cancers, some organ-specific impact of response was identified in various cancers [2, 3]. Notably, the immunotherapy-related studies of various cancers with liver metastases have shown unsatisfactory results [4, 5]. It has also been shown that the patients with liver metastases benefited less from immunotherapy and may be more likely to develop new metastatic lesions compared with other metastatic lesions such as lymph node metastases and lung metastases [3]. Some researchers think this phenomenon is associated with the unique tumor microenvironment in liver metastases.

It has been shown that high expression of PD-L1 in tumor was associated with poor prognosis [6–8] and that PD-L1-positive patients with lung cancer or esophageal cancer had a higher response rate to immunotherapy [9, 10]. The heterogeneity of PD-L1 expression may affect its prognostic and predictive accuracy. For instance, MASUGI et al. showed widespread heterogeneity of PD-L1 expression between centers and peripheral parts of primary tumors in colorectal cancer [11]. Studies on non-small cell lung cancer, endometrial cancer, gastric cancer and breast cancer showed that PD-L1 expressions were higher in metastases than in primary tumors [9, 12–14]. It is evident that exploring the differences in the immune microenvironment between the primary tumors and liver metastases is a key point to understand the reasons for the different responses to immunotherapy.

Colorectal cancer (CRC) is a very tricky malignancy worldwide [15]. [16] Autopsy suggested liver metastases in about 50% of colorectal cancer patients [17]. Unfortunately, in patients with liver metastases, the efficacy of regorafenib monotherapy or its combination with immunotherapy was undesirable [18, 19]. Previous studies have found the heterogeneity of lymphocyte type, lymphocyte number, KRAS status between primary tumors and liver metastases of colorectal cancer, which suggested that there might be difference in the microenvironment between primary tumors and liver metastases [20, 21].

The current study aim to explore the difference in PD-L1 expression status between primary tumors and liver metastases, and to find influence factors for PD-L1 expression disparity between primary tumors and liver metastases in CRC.

## Materials and methods

### Study group

Patients with pathologically confirmed CRC who underwent surgery for primary tumor and liver metastasis in Sun Yat-sen University Cancer Center were screened for this study. Initially, a total of 94 cases were considered. The clinical information about patient's age, sex, time of diagnosis, time of resection of metastases, location of primary tumor, metastasis pattern, tumor stage, tumor differentiation, situation of preoperative radiotherapy, situation of adjuvant treatment after resection of primary tumor and survival time, etc. were collected. The AJCC TNM staging system of the 8th edition was used for tumor staging [22]. A total of 74 patients were included in the final analysis by excluding 20 patients with incomplete clinical information.

### Tissue microarray (TMA) construction

An experienced pathologist was responsible for reviewing H&E-stained slides and marking the areas with abundant tumor cells to guide core selection. The donor tissue block was 4 mm thick and the recipient block was cast by melting conventional paraffin waxes in molds to make blank blocks. Then the donor tissue block was transferred into the recipient wax wells and prepared with a 0.6 mm perforated needle. Place the wax block in an incubator at 37 °C for 10 min so that the tissue core in the block and the wall of the pore were closely integrated. The wax block was then frozen on ice and sliced continuously to a thickness of 4 µm. It was used for immunohistochemistry (IHC) of the expression of PD-L1, cluster of differentiation 4 (CD4) and CD8 in CRC primary tumors and liver metastases.

### Immunohistochemistry

After finishing making the TMA blocks, bake the blocks in a 65 °C oven for 60 min, then cool to room temperature. The endogenous peroxidase was blocked by incubating with 3% hydrogen peroxide for 10 min after dewaxing and rehydration. Incubate primary antibodies (Rabbit antibodies for PD-L1 (SP142, spring bioscience), CD4(ZA-0508, ZSGBBIO), CD8(ZA-0519, ZSGB-BIO)) overnight at 4 °C in a refrigerator, wash with buffer and add the HRP RABBIT/MOUSE secondary antibody (K5007, 20,029,103, Dako) successively, then incubate at room temperature for 30 min. Then generally stain with diaminobenzidine tetrahydro-chloride (DAB, K5007, 20019193, Dako), stain with haematoxylin slightly for 1 min after color rendering was terminated.

### Evaluation of PD-L1 expression, CD4 and CD8 density

The results were interpreted by an experienced pathologist who read the images under a microscope and made comprehensive judgments. To determine the expression of PD-L1, the immunostaining of both tumor cells and tumor-infiltrating immune cells was considered, while the density of CD4 and CD8 was determined only for the staining of tumor-infiltrating immune cells. The Combined Positive Score (CPS) was used to evaluate PD-L1 expression levels. CPS ≥ 1 was defined as “PD-L1 expression positive” while CPS < 1 was defined as “PD-L1 expression negative”; As for CD4 and CD8 density, the proportion of cells < 10% was defined as “absent or low” while ≥ 10% was defined as “high”. To further identify the drivers of PD-L1 expression differences between primary tumor and liver metastases, the difference in CPS values of PD-L1 between primary and hepatic metastases of ≥ 5 was defined as "obvious discordance".

### Statistical analysis

The Statistical analysis software used in this study was SPSS for Windows V.13.0 (SPSS Inc., Chicago, IL, USA). The statistical methods for evaluating the relationship between PD-L1 expression and clinicopathological characteristics, CD4 density and CD8 density were chi-square test or Kruskal–Wallis H test. Consistency of PD-L1 expression in CRC was assessed using the chi-square test. Mann–Whitney U rank-sum test was used for the analysis of TNM staging of ordinal data. Comparisons of PD-L1 expression, CD4, and CD8 density in primary tumors versus liver metastases were performed with paired T test. Logistic regression analysis was used for the analysis of factors associated with obvious discordance of PD-L1 expression between primary tumors and liver metastases.

## Results

### Patient baseline characteristics

A total of 74 patients with CRC liver metastases were included in this study, 50 (67.6%) of whom were male and 24(32.4%) were female, with a median age of 56 years old ranging from 31 to 76 years old. The primary tumors of CRC was located in the proximal colon in 21 cases (28.4%), the distal colon in 19 cases (25.7%) and the rectum in 34 cases (45.9%). 63 patients (85.1%) had concurrent metastases and 18 (24.3%) had metachronous metastases, of which 66 (89.2%) had hepatic metastases only, and 8 cases (10.8%) had concomitant extrahepatic metastases. In terms of the tumor differentiation, 9 cases (12.2%) were poorly differentiated and 65 cases (87.8%) were moderately differentiated. 56 cases (75.5%) received concurrent resection of primary and metastatic liver tumors, and the remaining 18 cases (24.3%) received metachronous resection. Detailed information were shown in Additional file 1: Table S1.

### Clinicopathological factors associated with PD-L1 expression in liver metastases

**Fig. 1:**
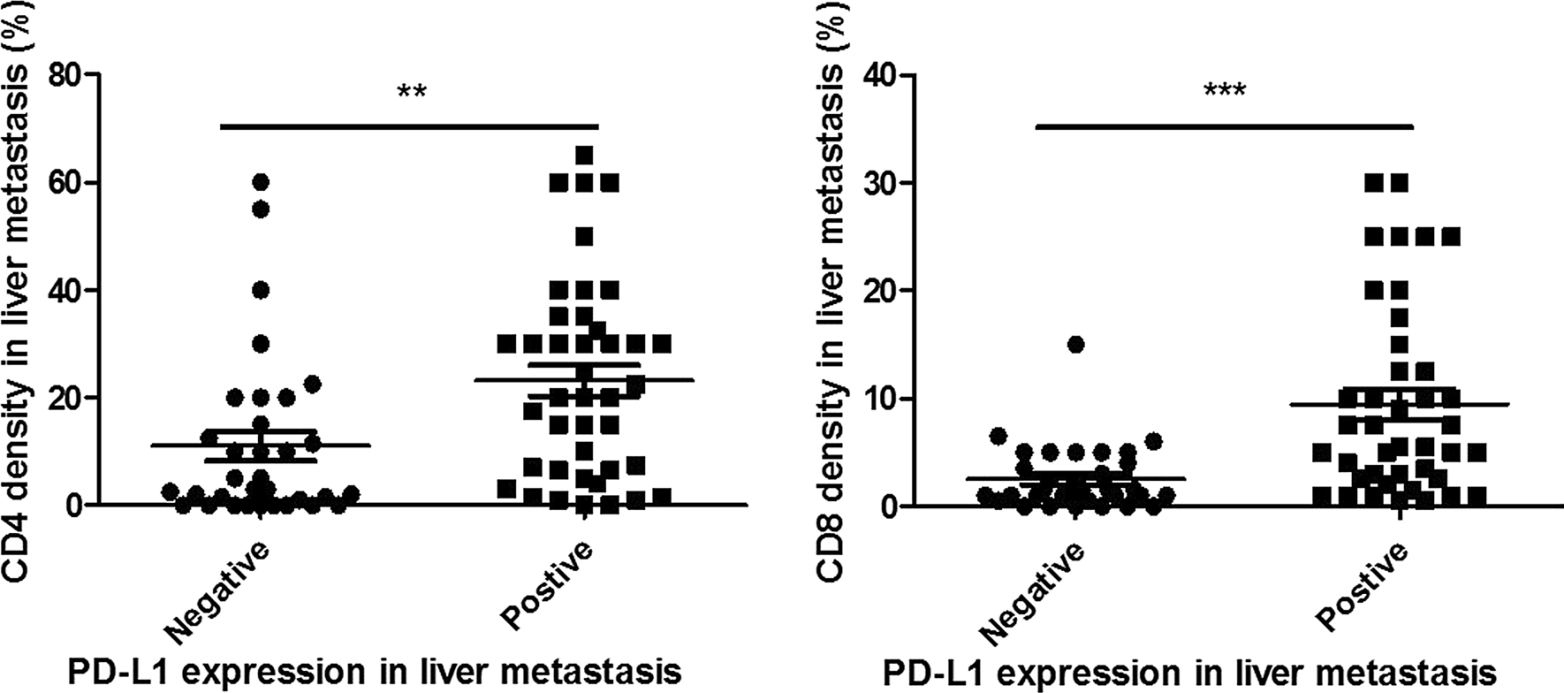
Association of PD-L1 expression with CD4 density and CD8 density in liver metastasis in CRC. Positive PD-L1 expression in liver metastasis is significantly related with higher levels of CD4 density and CD8 density in liver metastasis in CRC

**Fig. 2:**
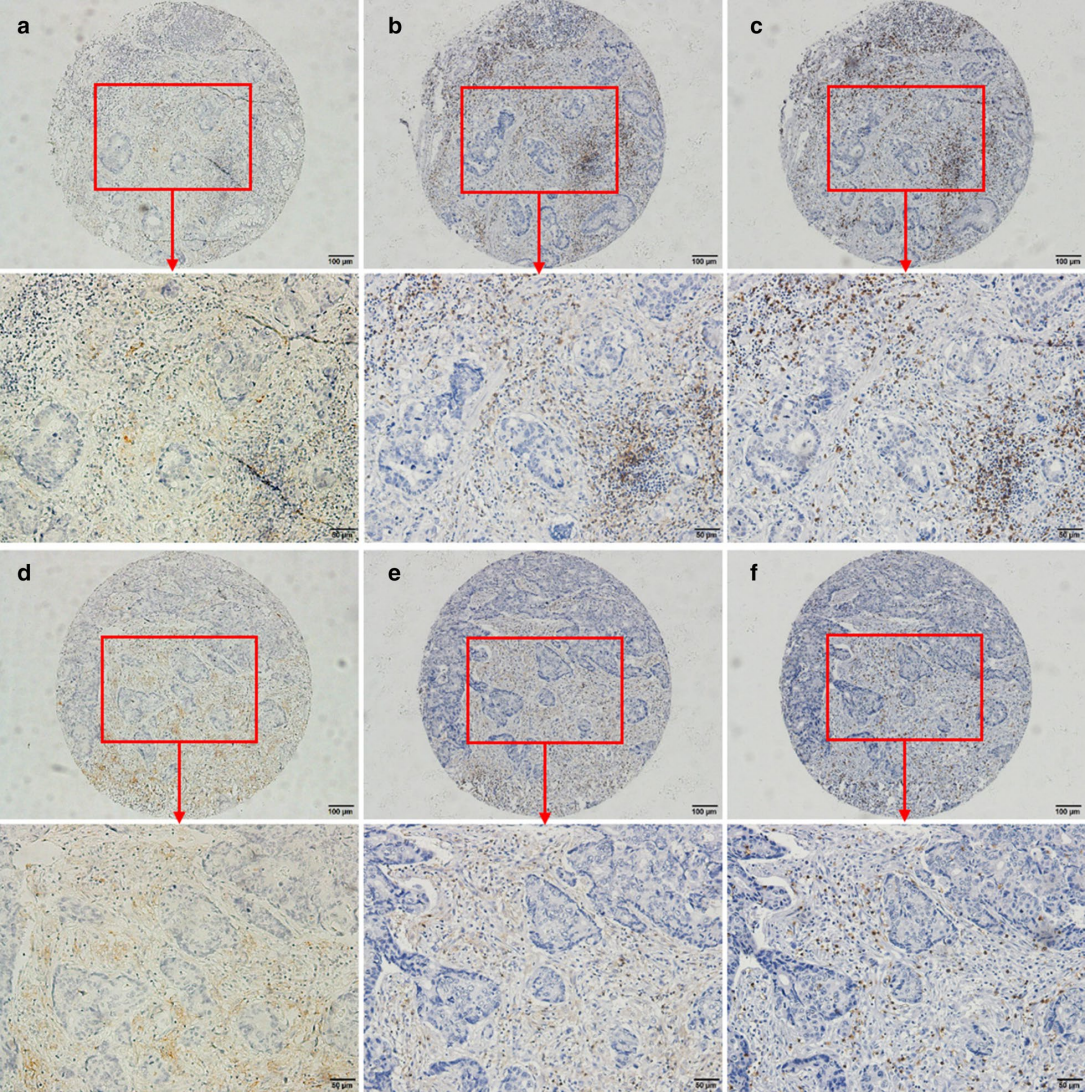
Representative immunostaining of PD-L1, CD4 and CD8 in primary tumor **a**–**c** and paired liver metastatic tumor (**d**–**f**). **a** PD-L1 expression in primary tumor with a CPS score of 5.5; **b** CD4 density in primary tumor with a percentage of 40%; **c** CD8 density in primary tumor with a percentage of 22.5%; **d** PD-L1 expression in liver metastasis with a CPS score of 20; **e** CD4 density in liver metastasis with a percentage of 40%; **f** CD8 density in liver metastasis with a percentage of 20%

According to Table 1, 41 out of 74 patients (55%) were positive for PD-L1 expression in liver metastases; The rate of PD-L1 positivity in rectal cancer liver metastases was higher than in colon cancer liver metastases (positivity rate:70.6% vs. 42.5%); The expression of PD-L1 in liver metastases was related to the density of CD4 and CD8 which were higher in PD-L1 positive patients (Fig. 1). 64.3% of PD-L1-positive patients have “high” CD4 density, while there was 35.7% in PD-L1-negative patients (*p* = 0.05); As for high density of CD8, the proportion (94.4%) for PD-L1-positive patients was higher than that (5.6%) for PD-L1-negative patients (*p* < 0.001). The patient's age, sex, whether there was concurrent metastasis and concurrent resection, TNM staging, tumor differentiation, and whether there was extrahepatic metastasis were not significantly correlated with the expression of PD-L1 in liver metastases. Representative immunostaining of PD-L1, CD4 and CD8 in primary tumor and liver metastasis is shown in Fig. 2.

**Table 1:** Clinicopathological factors associated with PD-L1 expression in liver metastasis

	PD-L1 expression^a^, N (%)		*P* value
	Negative	Positive	
Year of diagnosis			0.36
~ 2009	18 (50.0)	18 (50.0)	
2010 ~	15 (39.5)	23 (60.5)	
Age (years, median 56)			0.85
< 56	16 (45.7)	19 (54.3)	
≥ 56	17 (43.6)	22 (56.4)	
Gender			0.52
Male	21 (42.0)	29 (58.0)	
Female	12 (50.0)	12 (50.0)	
Primary tumor site			0.02*
Colon	23 (57.5)	17 (42.5)	
Rectum	10 (29.4)	24 (70.6)	
Metastatic time model			0.95
Concurrent	28 (44.4)	35 (55.6)	
Metachronous	5 (45.5)	6 (54.5)	
Resection time model			0.60
Concurrent	24 (42.9)	32 (57.1)	
Metachronous	9 (50.0)	9 (50.0)	
T category (AJCC TNM 8th)			0.31
T1 + T2	1 (16.7)	5 (83.3)	
T3	5 (45.5)	6 (54.5)	
T4	27 (47.4)	30 (52.6)	
N category (AJCC TNM 8th)			0.99
N0	6 (37.5)	10 (62.5)	
N1	10 (52.6)	9 (47.4)	
N2	11 (40.7)	16 (59.3)	
Nx	6 (50.0)	6 (50.0)	
Tumor differentiation			0.16
Poor	6 (66.7)	3 (33.3)	
Moderate	27 (41.5)	38 (58.5)	
Extra-hepatic metastasis			
No	28 (42.4)	38 (57.6)	
Yes	5 (62.5)	3 (37.5)	
CD4 density^b^ in liver metastatic tumor			0.05*
Absent and low	18 (56.3)	13 (43.8)	
High	15 (35.7)	28 (64.3)	
CD8 density^b^ in liver metastatic tumor			< 0.001*
Absent and low	32 (57.1)	24 (42.9)	
High	1 (5.6)	17 (94.4)	

*PD-L1* programmed death ligand 1, *N* number, *AJCC* American Joint Committee on Cancer, *TNM* tumor- node-metastasis, *CD4* cluster of differentiation 4, *CD8* cluster of differentiation 8

*Statistically significant

^a^Definition of PD-L1 expression: negative (CPS < 1), positive (CPS ≥ 1). *CPS* combined positive score. ^b^Definition of CD4, CD8 density: absent or low (< 10%), high (≥ 10%)

### Comparisons of PD-L1 expression, CD4 and CD8 density between primary tumors and liver metastases

**Fig. 3:**
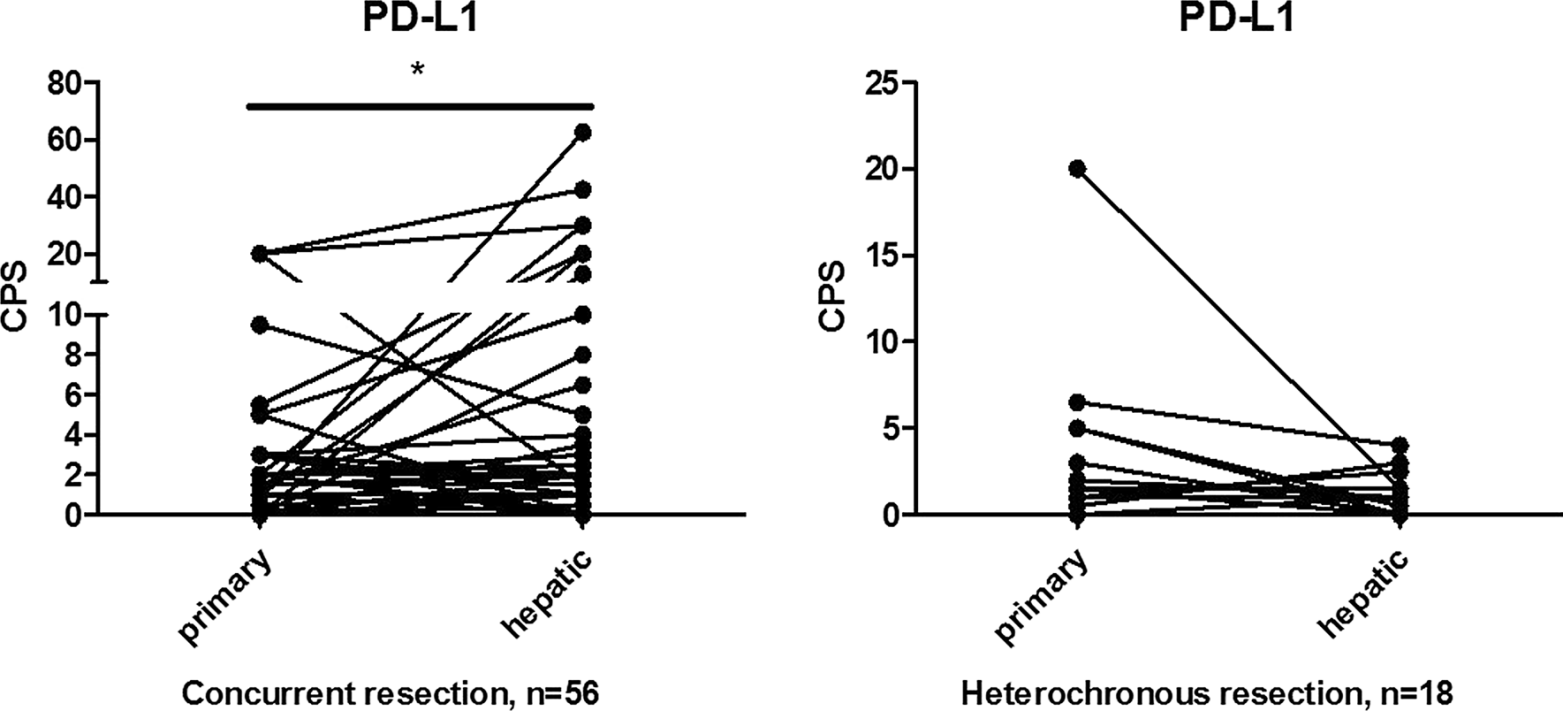
Comparisons of PD-L1 expression between primary tumors and liver metastasis in subgroups of CRC. PD-L1 expression (assessed using CPS scoring) was significantly higher in liver metastasis compared with primary colorectal tumors in the patients with concurrent resection of primary and metastatic tumors (n = 56) subgroup, but not in metachronous resection subgroup

The results (Table 2) showed that in terms of PD-L1 expression, although there was no significant difference in general PD-L1 expression between liver metastases and primary tumors (median: 1.0 vs. 0.5, *p* = 0.10), the CPS of PD-L1 was higher in liver metastases in patients with concurrent liver metastases (*p* = 0.05), and concurrent resection of primary tumor and liver metastases (*p* = 0.04, Fig. 3). The difference in PD-L1 expression between hepatic metastases and primary tumor was not statistically significant in patients who underwent chemo/radiotherapy for primary tumor.

**Table 2:** Comparisons of PD-L1 expression, CD4 and CD8 density between primary tumor and liver metastasis

Groups	PD-L1 CPS (median)		*P* value	CD4 density (%, median)		*P *value	CD8 density (%, median)		*P* value
	Primary tumor	Liver metastasis		Primary tumor	Liver metastasis		Primary tumor	Liver metastasis	
General	0.5	1.0	0.10	7.0	12.0	0.003*	4.5	3.5	0.63
Pre-operative che-mo/radiotherapy for primary tumor									
No	1.0	1.0	0.09	7.5	12.5	0.01*	4.0	5.0	0.78
Yes	0.0	0.0	0.31	5.5	7.5	0.28	5.0	1.5	0.03*
Tumor differentiation									
Poor	0.0	0.0	0.63	5.5	3.0	0.68	10.0	1.5	0.07
Moderate	0.5	1.0	0.052	7.5	15.0	0.003*	3.0	4.0	0.70
Primary tumor site									
Colon	0.0	0.0	0.27	5.3	10.0	0.03*	2.0	2.5	0.47
Rectum	1.5	1.3	0.20	10.0	17.5	0.04*	5.0	5.0	0.95
Metastatic time model									
Concurrent	0.0	1.0	0.05*	7.5	12.5	0.01*	5.0	3.5	0.95
Metachronous	1.5	1.0	0.22	3.0	5.0	0.22	2.0	4.0	0.32
Resection time model									
Concurrent	0.0	1.0	0.04*	8.8	15.0	0.01*	5.0	3.5	0.81
Metachronous	1.3	0.8	0.13	5.0	5.8	0.23	3.3	3.3	0.12

*PD-L1* programmed death ligand 1, *CPS* combined positive score, *CD4* cluster of differentiation 4, *CD8* cluster of differentiation 8

*Statistically significant

Overall, there were more CD4 + cells in liver metastases than in primary tumors (median of density: 12.0% vs. 7.0%, *p* = 0.003). In the subgroup analysis, the density of CD4 in liver metastases was significantly higher thanin primary tumors in patients with no radiotherapy before primary tumor resection (*p* = 0.01), concurrent liver metastases (*p* = 0.01), concurrent resection of primary tumors and hepatic metastases (*p* = 0.01).

In terms of CD8 density, under most circumstances, there was no significant difference between liver metastases and primary tumors. The density of CD8 in liver metastases was significantly lower than primary tumors in those underwent chemo/radiotherapy before primary tumors resected (median: 1.5% vs. 5.0%, *p* = 0.03).

### Logistic regression analysis for factors associated with an obvious discordance of PD-L1 expression between primary tumors and liver metastatic tumors

Detailed data on the obvious discordance of PD-L1 expression between liver metastases and primary tumors are shown in Additional file 1: Table S2. The results of the logistic regression analysis of the relevant factors are shown in Table 3. The results showed that tumor differentiation (moderate vs. poor: *OR* = 0.23, 95% *CI*: 0.03–0.99, *p* = 0.05), discordance of the density of CD8 between primary tumors and liver metastases (yes vs. no: *OR* = 8.95, 95% *CI*: 2.06–39.00, *p* = 0.004) were associated with obvious discordance of PD-L1 expression between primary tumors and liver metastases. Whether the liver metastases were concurrent, and whether the primary tumors were resected concurrently with the liver metastases were not associated with the obvious discordance.

**Table 3:** Logistic regression analysis for factors associated with an obvious discordance of PD-L1 expression between primary tumors and liver metastatic tumors

	Obvious discordance of PD-L1 expression^a^, N. (%)		Univariate analysis		*P*-value	Multivariate analysis		*P* value
	No (n = 60)	Yes (n = 14)	OR	95% CI		AOR	95% CI	
Year of diagnosis					0.29			
~ 2009	31 (86.1)	5 (13.9)	1	Reference				
2010 ~	29 (76.3)	9 (23.7)	1.92	0.58–6.42				
Age (years, median 56)					0.42			
< 56	27 (77.1)	8 (22.9)	1	Reference				
≥ 56	33 (84.6)	6 (15.4)	0.61	0.19–1.99				
Gender					0.12			
Male	38 (76.0)	12 (24.0)	1	Reference				
Female	22 (91.7)	2 (8.3)	0.29	0.06–1.41				
Primary tumor site					0.35			
Colon	34 (85.0)	6 (15.0)	1	Reference				
Rectum	26 (76.5)	8 (23.5)	1.74	0.54–5.65				
Metastatic time model					0.38			
Concurrent	50 (79.4)	13 (20.6)	1	Reference				
Metachronous	10 (90.0)	1 (9.1)	0.39	0.05–3.28				
Resection time model					0.34			
Concurrent	44 (78.6)	12 (21.4)	1	Reference				
Metachronous	16 (88.9)	2 (11.1)	0.46	0.09–2.28				
T category (AJCC TNM 8^th^)					0.79			
T1 + T2	4 (66.7)	2 (33.3)	1	Reference				
T3	11 (100.0)	0 (0.0)	< 0.001	< 0.001-NA	1.00			
T4	45 (78.9)	14 (18.9)	0.53	0.09–3.27	0.50			
N category (AJCC TNM 8^th^)					0.19			
N0	10 (62.5)	6 (37.5)	1	Reference				
N1	17 (89.5)	2 (10.5)	0.20	0.03–1.16	0.07			
N2	22 (81.5)	5 (18.5)	0.38	0.09–1.54	0.18			
Nx	11 (91.7)	1 (8.3)	0.15	0.02–1.49	0.11			
Tumor differentiation					0.05*			0.05*
Poor	5 (55.6)	4 (44.4)	1	Reference		1	Reference	
Moderate	55 (84.6)	10 (15.4)	0.23	0.05–1.00		0.18	0.03–0.99	
Extra-hepatic metastasis					0.63			
No	53 (80.3)	13 (19.7)	1	Reference				
Yes	7 (87.5)	1 (12.5)	0.58	0.07–5.16				
Pre-operative chemo/radiotherapy for primary tumor					0.28			
No	48 (78.7)	13 (21.3)	1	Reference				
Yes	12 (92.3)	1 (7.7)	0.31	0.04–2.59				
Discordance of CD4 density between primary and metastatic tumor					0.86			
No	40 (81.6)	9 (18.4)	1	Reference				
Yes	20 (80.0)	5 (20.0)	1.11	0.32–3.76				
Discordance of CD8 density between primary and metastatic tumor					0.003*			0.004*
No	41 (93.2)	3 (6.8)	1	Reference		1	Reference	
Yes	19 (63.3)	11 (36.7)	7.91	1.98–31.69		8.95	2.06–39.00	

*N* number, *OR* odds ratio, *CI* confidence interval, *AOR* adjusted odds ratio, *PD-L1* programmed death ligand 1, *AJCC* American Joint Committee on Cancer, *TNM* tumor- node-metastasis, *CD4* cluster of differentiation 4, *CD8* cluster of differentiation 8

*Statistically significant

^a^Defined as a CPS score gap between primary tumor and liver metastatic tumor ≥ 5

## Discussion

The effect of immunotherapy for liver metastases of various tumors remain unsatisfactory [4]. In CRC, EPOC1603 clinical trial of regorafenib plus nivolumab therapy showed promising effect with an ORR of 36% in treatment-refractory CRC, but relatively unsatisfactory outcomes was found in the subgroup of patients with liver metastases [18]. Meanwhile, phase IIIb CONSIGN study showed that regorafenib significantly improved survival in treatment-refractory CRC, but liver metastases was a significant adverse factor for progression-free survival (PFS) [19]. Our results suggest that there are differences in microenvironment between the liver metastases and the primary tumors, and that the immunosuppressive status of liver metastases is more pronounced, which may be one important reason for the poor effect of immunotherapy in colorectal cancer with liver metastases.

The poor outcome of patients with liver metastases suggested a possibly distinct microenvironment in liver metastases. Previous researches have provided several possible explanations for this phenomenon. The results of TUMEH’s study showed that CD8 + T cells, which are important effector cells for anti-PD-1/PD-L1 therapy, were significantly reduced at the margin of metastatic lesions in patients with liver metastases compared to patients without liver metastases. [23] Researchers believed this may be related to the liver tolerance. Liver tolerance mechanisms considered that, the liver, as an important immune organ, is exposed to a large quantity of antigens from the gastrointestinal tract and the portal system [24, 25]. On the one hand, there are a large number of immune cells in the liver that can activate the immune response against pathogens that may harm the organism rapidly, and on the other hand, the liver needs to suppress the overreaction of the immune system through certain mechanisms in order to maintain the stability of the internal environment [24, 25]. This tolerance may be related to incomplete CD8 + T-cell activation or effector T-cell inactivation, CD4 + T-cell inactivation and regulatory T-cell activation induced by Kupffer cells [26]. In our study, a higher PD-L1 level in liver metastases suggested that PD-L1 expression might also be involved in liver tolerance. However, combining the relative resistance to PD-1/PD-L1 inhibitors in patients with liver metastasis, we proposed that the ability of PD-1/PD-L1 inhibitors to enhance the immune system's response may be partially counteracted by other mechanisms of liver tolerance, thus compromise the effectiveness of treatment [27].

In the present study, we found that PD-L1 expression was higher in certain subgroups of metastases, which included patients who did not receive chemotherapy during the interval of resection of primary tumor and liver metastasis, patients with concurrent liver metastasis and patients with concurrent resection of primary tumor and liver metastasis. All those subgroups were patients whose primary tumors and liver metastases were not impacted by inconsistent external influencing factors, especially treatment and temporal factors. And these subgroups were suitable for analysis of the intrinsic differences of microenvironment between primary tumors and liver metastases. Our results suggested that liver metastases had higher expression of PD-L1 than primary tumors and were in a more pronounced immunosuppressive status in the absence of external factors such as chemotherapy and temporal variation. This heterogeneity in PD-L1 expression was consistent with the results of WANG et al. on 22 patients with CRC with metastasis whose metastatic lesion had higher PD-L1 expression than their primary tumor [28]. Meanwhile, researches on non-small cell lung cancer, endometrial cancer, breast cancer, etc., had similar results [9, 12, 14].

We also found that the proportion of CD8 + cells was higher in primary tumors than in metastatic lesion when the patients had received radiotherapy or chemotherapy before resection of primary tumors, suggesting that radiotherapy or chemotherapy may enhance the infiltration of CD8 + cells in primary tumor. At the same time, we found that the level of PD-L1 expression in rectal cancer liver metastases was higher than that in colon cancer liver metastases, which might be related to the fact that some patients with rectal cancer had undergone chemoradiotherapy. HUANG et al. also showed that neoadjuvant chemotherapy with decitabine promoted expression of immune-related gene and proliferation of TILs [29]. However, whether radiotherapy/ chemotherapy enhanced CD8 + T cells infiltration and whether it could improve the response rates to immunotherapy remains to be researched in more trials.

We further analyzed the factors related to the discordance in PD-L1 expression using CPS difference ≥ 5 as the boundary. The results showed that only the tumor differentiation and the discordance in the density of CD8 between primary tumors and liver metastases were associated with this discordance. Literature reported that poorly differentiated tumors may contain more cancer stem cells (CSC), a type of cell with specific biological properties such as self-renewal and differentiation potential [30]. The CSC model has long been considered as an important mechanism leading to phenotypic and functional heterogeneity and generating tumor diversity, tumors with more CSCs can evolve into stronger heterogeneous tumors. In terms of spatial and temporal heterogeneity of the tumor, there was heterogeneity in the microenvironment in different regions of the same tumor, mainly in terms of differences in oxygen availability, acidity, nutrient availability, and lymphocyte infiltration within the tumor [31, 32]. Poorly differentiated tumors have been shown to have higher microvascular and microlymphatic densities than well-differentiated tumors [33, 34]. These heterogeneities were likely to influence the tumor PD-L1 expression leading to the obvious discordance between primary tumors and metastatic lesions observed in this study. In combination with the increased density of CD8 in the PD-L1-positive group in the hepatic metastases, we considered that the discordance of CD8 between primary tumor and liver metastases should be a concomitant state of differential PD-L1 expression, and that the tumor differentiation which is closely related to tumor heterogeneity may be the intrinsic driver of this discordance. PD-L1 expression had been proposed to be a biomarker for benefit from PD-1/PD-L1 inhibitors in several cancers. While its inter-tumor spatially heterogeneous expression had been recognized and affected its reliability [9, 13]. The present study suggested that PD-L1 expression of patients with poor tumor differentiation may more likely need to be tested for both primary tumors and metastatic lesions.

As the main tumor-infiltrating lymphocytes, CD8 + T cell was the reaction center for the alternative mechanism, and the key cell for immunotherapy such as anti-PD-L1 therapy. Our results showed an increased number of CD8 + infiltrating cells in PD-L1-positive individuals with liver metastases. However, ZHOU et al. studied 44 CRC patients with liver metastases and found that most of the immune cells in liver metastases were CD33 + inhibitory immune cells, and most of the CD8 + cells were not CD8 + T cells [20]. Moreover, large numbers of suppressive immune cells promote aggregation of myeloid-derived suppressor cells (MDSCs) in hepatic metastases, which can inhibit the proliferation of actived T cell but promote proliferation of suppressor T cell, and also promote tumor angiogenesis, invasion, and metastasis, and reduce the efficacy of immunotherapy [35]. In addition, TOOR et al. suggested that CD4 + lymphocytes in CRC tumor tissues are predominantly regulatory T cells (TREG) which can promote expression of immune checkpoints including cytotoxic T-lymphocyte-associated protein-4 (CTLA-4), T cell immunoglobulin and mucin domain-3 (TIM-3), and lymphocyte-activated gene 3 (LAG-3), then further promote immune escape in cancer cells [36]. SHITARA et al. also found that TREG could inhibit anti-tumor immunity effect of cells by inhibiting the effect of antigen-producing cells and secreting inhibitory cytokines such as TGF-B, IL-10 and IL-35 to inhibit the function of effector T cells or promote apoptosis of effector T cells [37]. In this study, 58% (43/74) of patients had “high” CD4 expression in liver metastatic lesions and 64.3% in PD-L1-positive group; Table 3 also showed that the proportion of CD4 + lymphocytes in liver metastatic lesions was significantly higher than in primary tumors (12.0% vs. 7.0%). Whether the majority of CD4 + lymphocytes in liver metastases were TREG and thus affected the efficacy of immunotherapy requires further subgroup analysis of CD4 + cells in the future.

Although this study has the highest number of cases included among studies analyzing the difference in PD-L1 expression between primary tumors and liver metastases of CRC, it still has some limitations. Firstly, this was a retrospective study, and the cases included were surgically resected with few patients having to be excluded due to lack of clinical information, inevitably leading to selection bias. The patients we selected received resection for liver metastases, which were generally small and had a low tumor load, and this may cause this study not to reflect the circumstance of larger liver metastases or greater tumor burden. In addition, we did not further study the subgroups of CD4 + and CD8 + cells in the tumor immune microenvironment and failed to further explore the detailed mechanisms of immunosuppression in liver metastases. More detailed studies about the expression of immune checkpoints in the primary center and peripheral parts of the tumors, including comparison of immune cell subgroups, are still needed to be conducted in the future. Last but not least, we used TMAs to evaluate PD-L1 expression, which may result in certain bias due to intra-tumor heterogeneity of immune microenvironment [38]. Our results need to be confirmed with immunostaining of whole sections.

## Conclusion

In conclusion, in the present study, we not only elucidated the expression pattern of PD-L1 in CRC between primary tumors and liver metastases, but also identified tumor differentiation as a causal factor for the obvious discordance of PD-L1 expression between primary tumors and liver metastases.

## Supplementary Information

**Additional file 1: Table S1.** Baseline characteristics of patients. **Table S2.** Patients with obvious PD-L1 discordant expression between primary and metastatic tumor.

## Data Availability

All data generated or analysed during this study are included in this published article and its supplementary information files. Other data that were not relevant for the results presented here are available from the corresponding author Dr. Xu on reasonable request.

## Abbreviations

PD-L1: Programmed cell death ligand-1
CRC: Colorectal cancer
TMA: Tissue microarray
IHC: Immunohistochemistry
CD4: Cluster of differentiation 4
CD8: Cluster of differentiation 8
CPS: Combined Positive Score
CSC: Cancer stem cells

## References

[1] Wei SC, Duffy CR, Allison JP (2018). Fundamental mechanisms of immune checkpoint blockade therapy. Cancer Discov.

[2] Osorio JC, Arbour KC, Le DT, Durham JN, Plodkowski AJ, Halpenny DF (2019). Lesion-level response dynamics to programmed cell death protein (PD-1) blockade. J Clin Oncol.

[3] Silva IPD, Lo S, Quek C, Gonzalez M, Carlino MS, Long GV (2020). Site-specific response patterns, pseudoprogression, and acquired resistance in patients with melanoma treated with ipilimumab combined with anti-PD-1 therapy. Cancer-Am Cancer Soc.

[4] Botticelli A, Cirillo A, Scagnoli S, Cerbelli B, Strigari L, Cortellini A (2020). The agnostic role of site of metastasis in predicting outcomes in cancer patients treated with immunotherapy. Vaccines.

[5] Bilen MA, Shabto JM, Martini DJ, Liu Y, Lewis C, Collins H (2019). Sites of metastasis and association with clinical outcome in advanced stage cancer patients treated with immunotherapy. Bmc Cancer.

[6] Jung HI, Jeong D, Ji S, Ahn TS, Bae SH, Chin S (2017). Overexpression of PD-L1 and PD-L2 is associated with poor prognosis in patients with hepatocellular carcinoma. Cancer Res Treat.

[7] Webba JR, Milnea K, Kroegera DR, Nelson BH (2016). PD-L1 expression is associated with tumor-infiltrating T cells and favorable prognosis in high-grade serous ovarian cancer. Gynecol Oncol.

[8] Fang W, Chen Y, Sheng J, Zhou T, Zhang Y, Zhan J (2017). Association between PD-L1 expression on tumour-infiltrating lymphocytes and overall survival in patients with gastric cancer. J Cancer.

[9] Hong L, Negrao MV, Dibaj SS, Chen R, Reuben A, Bohac JM (2020). Programmed death ligand 1 heterogeneity and its impact on benefit from immune checkpoint inhibitors in non-small-cell lung cancer. J Thorac Oncol.

[10] Huang T, Fu L (2019). The immune landscape of esophageal cancer. Cancer Commun (London, England).

[11] Masugi Y, Nishihara R, Yang J, Mima K, Silva AD, Shi Y (2017). Tumour cd274 (PD-L1) expression and T cells in colorectal cancer. Gut.

[12] Engerud H, Berg HF, Myrvold M, Halle MK, Bjorge L, Haldorsen IS (2020). High degree of heterogeneity of PD-L1 and PD-1 from primary to metastatic endometrial cancer. Gynecol Oncol.

[13] He PX, Ma ZL, Han H, Zhang XY, Niu SH, Du LN (2020). Expression of programmed death ligand 1 (PD-L1) is associated with metastasis and differentiation in gastric cancer. Life Sci.

[14] Li M, Li A, Zhou S, Xu Y, Xiao Y, Bi R (2018). Heterogeneity of PD-L1 expression in primary tumors and paired lymph node metastases of triple negative breast cancer. BMC Cancer.

[15] Dekker E, Tanis PJ, Vleugels JLA, Kasi PM, Wallace MB (2019). Colorectal cancer. Lancet (London, England).

[16] Feng RM, Zong YN, Cao SM, Xu RH (2019). Current cancer situation in china: good or bad news from the 2018 global cancer statistics?. Cancer Commun (Lond).

[17] Xu J, Fan J, Qin X, Cai J, Gu J, Wang S (2019). Chinese guidelines for the diagnosis and comprehensive treatment of colorectal liver metastases (version 2018). J Cancer Res Clin.

[18] Fukuoka S, Hara H, Takahashi N, Kojima T, Kawazoe A, Asayama M (2020). Regorafenib plus nivolumab in patients with advanced gastric or colorectal cancer: an open-label, dose-escalation, and dose-expansion phase Ib trial (regonivo, epoc1603). J Clin Oncol.

[19] Van Cutsem E, Martinelli E, Cascinu S, Sobrero A, Banzi M, Seitz JF (2019). Regorafenib for patients with metastatic colorectal cancer who progressed after standard therapy: results of the large, single-arm, open-label phase IIIb consign study. Oncologist.

[20] Zhou SN, Pan WT, Pan MX, Luo QY, Zhang L, Lin JZ (2020). Comparison of immune microenvironment between colon and liver metastatic tissue in colon cancer patients with liver metastasis. Dig Dis Sci.

[21] Ekinci AS, Demirci U, Oksuzoglu BC, Ozturk A, Esbah O, Ozatli T (2015). Kras discordance between primary and metastatic tumor in patients with metastatic colorectal carcinoma. J BUON.

[22] Mr W (2018). AJCC 8th edition: colorectal cancer. Ann Surg Oncol.

[23] Tumeh PC, Hellmann MD, Hamid O, Tsai KK, Loo KL, Gubens M (2017). Liver metastasis and treatment outcome with anti-PD-1 monoclonal antibody in patients with melanoma and NSCLC. Cancer Immunol Res.

[24] Heymann F, Tacke F (2016). Immunology in the liver–from homeostasis to disease. Nat Rev Gastroenterol Hepatol.

[25] Morisa D, Lua L, Qian S (2017). Mechanisms of liver-induced tolerance. Curr Opin Organ Tran.

[26] Qin B, Jiao X, Liu J, Liu K, He X, Wu Y (2020). The effect of liver metastasis on efficacy of immunotherapy plus chemotherapy in advanced lung cancer. Crit Rev Oncol Hemat.

[27] Li S, Sun S, Xiang H, Yang J, Peng M, Gao Q (2020). Liver metastases and the efficacy of the PD-1 or PD-L1 inhibitors in cancer: a meta-analysis of randomized controlled trials. Oncoimmunology.

[28] Wang HB, Yao H, Li CS, Liang LX, Zhang Y, Chen YX (2017). Rise of PD-L1 expression during metastasis of colorectal cancer: implications for immunotherapy. J Digest Dis.

[29] Huang KC, Chiang S, Chen WT, Chen T, Hu C, Yang P (2020). Decitabine augments chemotherapy-induced PD-L1 upregulation for PD-L1 blockade in colorectal cancer. Cancers.

[30] Tang DG (2012). Understanding cancer stem cell heterogeneity and plasticity. Cell Res.

[31] Meacham CE, Morrison SJ (2013). Tumour heterogeneity and cancer cell plasticity. Nature.

[32] Jögiab A, Vaapilab M, Johanssona M, Påhlman S (2012). Cancer cell differentiation heterogeneity and aggressive behavior in solid tumors. Upsala J Med Sci.

[33] Xie B, Wang Y, He J, Ni Z, Chai D (2019). Aberrant cyclin e and hepatocyte growth factor expression, microvascular density, and micro-lymphatic vessel density in esophageal squamous cell carcinoma. Cancer Control.

[34] Sundov Z, Tomic S, Alfirevic S, Sundov A, Capkun V, Nincevic Z (2013). Prognostic value of MVD, LVD and vascular invasion in lymph node-negative colon cancer. Hepatogastroenterology.

[35] Chesney JA, Mitchell RA, Yaddanapudi K (2017). Myeloid-derived suppressor cells-a new therapeutic target to overcome resistance to cancer immunotherapy. J Leukocyte Biol.

[36] Toor SM, Murshed K, Al-Dhaheri M, Khawar M, Nada AM (2019). Immune checkpoints in circulating and tumor-infiltrating CD4 T cell subsets in colorectal cancer patients. Front Immunol.

[37] Shitara K, Nishikawa H (2018). Regulatory T cells: a potential target in cancer immunotherapy. Ann Ny Acad Sci.

[38] Botti G, Scognamiglio G, Cantile M (2016). PD-L1 immunohistochemical detection in tumor cells and tumor microenvironment: main considerations on the use of tissue micro arrays. Int J Mol Sci.

