# Supplementary material for: PD-L1 expression in liver metastasis: its clinical significance and discordance with primary tumor in colorectal cancer
Source: J Transl Med. 2020 Dec 11;18:475. doi: 10.1186/s12967-020-02636-x (PMC7730753; doi:10.1186/s12967-020-02636-x)
Supplement: Supplementary file 1 — Additional file 1: Table S1. Baseline characteristics of patients. Table S2. Patients with obvious PD-L1 discordant expression between primary and metastatic tumor. [file 12967_2020_2636_MOESM1_ESM.docx]

Supplementary table 1. Baseline characteristics of patients.

|  | N. (%), total: 74 |
| --- | --- |
| Year of diagnosis |  |
| ~2009 | 36 (48.6) |
| 2010~ | 38 (51.4) |
| Age (median, range) | 56 (31-76) |
| Gender |  |
| Male | 50 (67.6) |
| Female | 24 (32.4) |
| Primary tumor site |  |
| Proximal colon | 21 (28.4) |
| Distal colon | 19 (25.7) |
| Rectum | 34 (45.9) |
| Metastatic time model |  |
| Concurrent | 63 (85.1) |
| Metachronous | 11 (14.9) |
| Resection time model |  |
| Concurrent | 56 (75.7) |
| Metachronous | 18 (24.3) |
| T category (AJCC TNM 8^th^ ) |  |
| T1+T2 | 6 (8.1) |
| T3 | 11 (14.9) |
| T4 | 57 (77.0) |
| N category (AJCC TNM 8^th^ ) |  |
| N0 | 16 (21.6) |
| N1 | 19 (25.7) |
| N2 | 27 (36.5) |
| Nx | 12 (16.2) |
| Tumor differentiation |  |
| Poor | 9 (12.2) |
| Moderate | 65 (87.8) |
| Extra-hepatic metastasis |  |
| No | 66 (89.2) |
| Yes | 8 (10.8) |

Abbreviations: N, number; AJCC, American Joint Committee on Cancer; TNM, tumor- node-metastasis.

Supplementary table 2. Patients with obvious PD-L1 discordant expression between primary and metastatic tumor.

| Sequence number | PD-L1 expression (CPS score) | | CD8 density (%) | | Tumor differentiation |
| --- | --- | --- | --- | --- | --- |
|  | Primary tumor | Metastatic tumor | Primary tumor | Metastatic tumor |  |
| 1 | 5.5 | 20.0 | 22.5 | 20.0 | 2 |
| 2 | 5.0 | .0 | 15.0 | 1.0 | 1 |
| 3 | 5.0 | 10.0 | .0 | 10.0 | 2 |
| 4 | 20.0 | 30.0 | 15.0 | 30.0 | 2 |
| 5 | 2.0 | 30.0 | 1.0 | 12.5 | 2 |
| 6 | 20.0 | 1.5 | 30.0 | 5.0 | 1 |
| 7 | .0 | 8.0 | 1.0 | 10.0 | 2 |
| 8 | 20.0 | 1.5 | 1.0 | 7.5 | 2 |
| 9 | 1.0 | 62.5 | 8.0 | 2.0 | 1 |
| 10 | 20.0 | 42.5 | 3.0 | 1.0 | 2 |
| 11 | 5.0 | .0 | 15.0 | 1.5 | 1 |
| 12 | 1.0 | 13.0 | 10.0 | 3.0 | 2 |
| 13 | 1.5 | 6.5 | 15.0 | 5.0 | 2 |
| 14 | .0 | 20.0 | 15.5 | 25.0 | 2 |

Abbreviations: PD-L1, programmed death ligand 1; CPS, combined positive score.
